# Long-Term Safety of Mometasone Furoate/Formoterol Combination for Treatment of Patients with Persistent Asthma

**DOI:** 10.3109/02770903.2010.514634

**Published:** 2010-09-28

**Authors:** Jorge F Maspero, Hendrik Nolte, Iván Chérrez-Ojeda

**Affiliations:** 1 Fundacion CIDEA, Allergy/Respiratory Research, Buenos Aires, Argentina; 2 Merck Research Laboratories, Kenilworth, New Jersey, USA; 3 RESPIRALAB Allergy, Respiratory & Sleep Center, Guayaquil, Ecuador

**Keywords:** asthma, formoterol, long-term safety, metered-dose inhaler, mometasone furoate, mometasone furoate/formoterol

## Abstract

*Objective:* The combination of inhaled corticosteroid (ICS) and long-acting β_2_-agonist is recommended for treatment of patients with persistent asthma inadequately controlled on ICS monotherapy. This study was conducted to evaluate the long-term safety of mometasone furoate/formoterol (MF/F) administered through metered-dose inhaler (MDI) in patients with persistent asthma previously on medium- to high-dose ICS. *Methods:* This was a 52-week, randomized, multicenter, parallel-group, open-label, evaluator-blinded study. At baseline, 404 patients (aged >12 years) were stratified according to their previous ICS dose (medium or high), then randomized 2:1 to receive twice-daily treatment of MF/F (200/10 or 400/10 μg) or fluticasone propionate/salmeterol (FP/S; 250/50 or 500/50 μg). The primary endpoint was the number and percentage of patients reporting any adverse event (AE). Additional safety evaluations included plasma cortisol 24-hour area under the curve (AUC_0–24h_) and ocular changes. Pulmonary function, asthma symptoms, and use of rescue medication were monitored. *Results:* The incidence of >1 treatment-emergent AE was similar across treatment groups (MF/F 200/10 μg, 77.3% [*n* = 109]; FP/S 250/50 μg, 82.4% [*n* = 56]; MF/F 400/10 μg, 79.2% [*n* = 103]; FP/S 500/50 μg, 76.9% [*n* = 50]). Rates of treatment-related AEs were also similar across treatment groups (MF/F 200/10 μg, 28.4%; FP/S 250/50 μg, 23.5%; MF/F 400/10 μg, 23.1%; FP/S 500/50 μg, 20.0%). Headache (3.7%) and dysphonia (2.7%) were the most common treatment-related AEs overall. The nature and frequency of AEs and the decreases in plasma cortisol AUC_0–24 h_ observed with MF/F treatment were similar to those observed with FP/S treatment. Ocular events were rare (2–6% overall incidence among treatment groups); in particular, no posterior subcapsular cataracts were reported. Only three patients discontinued the study because of treatment-related ocular AEs (two for lens disorders in the MF/F 400/10 μg group; one for reduced visual acuity in the FP/S 250/50 μg group) and no asthma-related deaths occurred. Furthermore, MF/F showed numerical improvement in lung function and clinical benefits by reducing asthma symptoms and rescue medication use. *Conclusions:* One-year treatment with the new combination therapies -twice-daily MF/F-MDI 200/10 and 400/10 μg — is safe and well tolerated in patients with persistent asthma.

## Introduction

Asthma is a chronic respiratory disease characterized by reversible bronchoconstriction, bronchial hyperrespon-siveness, and inflammation, with episodes of asthma worsening occurring in response to various stimuli (1–4). With their anti-inflammatory properties, inhaled cortico-steroids (ICSs) are the first-line therapy to relieve persistent asthma symptoms of all severities (1–4). For treatment of patients with moderate to severe asthma who are not adequately controlled on ICS monotherapy or whose disease severity warrants treatment with an additional controller medication, combination therapy with an ICS and a bronchodilator (e.g., long-acting β2-agonist [LABA]) is recommended by asthma guidelines (5, 6).

Mometasone furoate (MF), a potent and safe ICS with high affinity for glucocorticoid receptors, is approved in the United States for treatment of asthma in adult and pediatric patients (7). Numerous clinical trials have demonstrated the efficacy of MF with regard to lung function, asthma symptoms, and quality of life as well as its safety and tolerability at multiple strengths (100–800 μg) and dosing regimens (8–15). Formoterol (F), a LABA, when administered as monotherapy, rapidly dilates airway smooth muscle and maintains control over 24 hours, thereby improving lung function and reducing asthma symptoms (16–19).

Because individual components of MF/F possess well-defined efficacy as well as pharmacologic and safety profiles when administered separately at recommended doses, the new MF/F combination administered through a single metered-dose inhaler (MDI) is expected to exhibit the same characteristics. A 52-week study was undertaken to determine the long-term safety, including cortisol suppression and ocular changes, of MF/F in patients with persistent asthma previously on maintenance therapy of medium- to high-dose ICS alone or in combination with a LABA. In addition, the study monitored pulmonary function, asthma symptom scores, rescue medication use, and asthma exacerbations to ensure appropriate disease management. Fluticasone propionate plus salmeterol (FP/S) MDI, a frequently prescribed ICS/LABA combination, was selected as the active comparator (20, 21).

## Methods

### Study Design

This was a 52-week, randomized, parallel-group, mul-ticenter, open-label, and evaluator-blinded (third-party study medication dispenser) study conducted at 27 clinical sites in South America (Argentina, Peru, Ecuador, Guatemala, Chile, and Mexico) in compliance with Good Clinical Practice guidelines. The study protocol was reviewed and approved by institutional review boards. Written informed consent was obtained from all patients before study entry.

**Figure 1:**
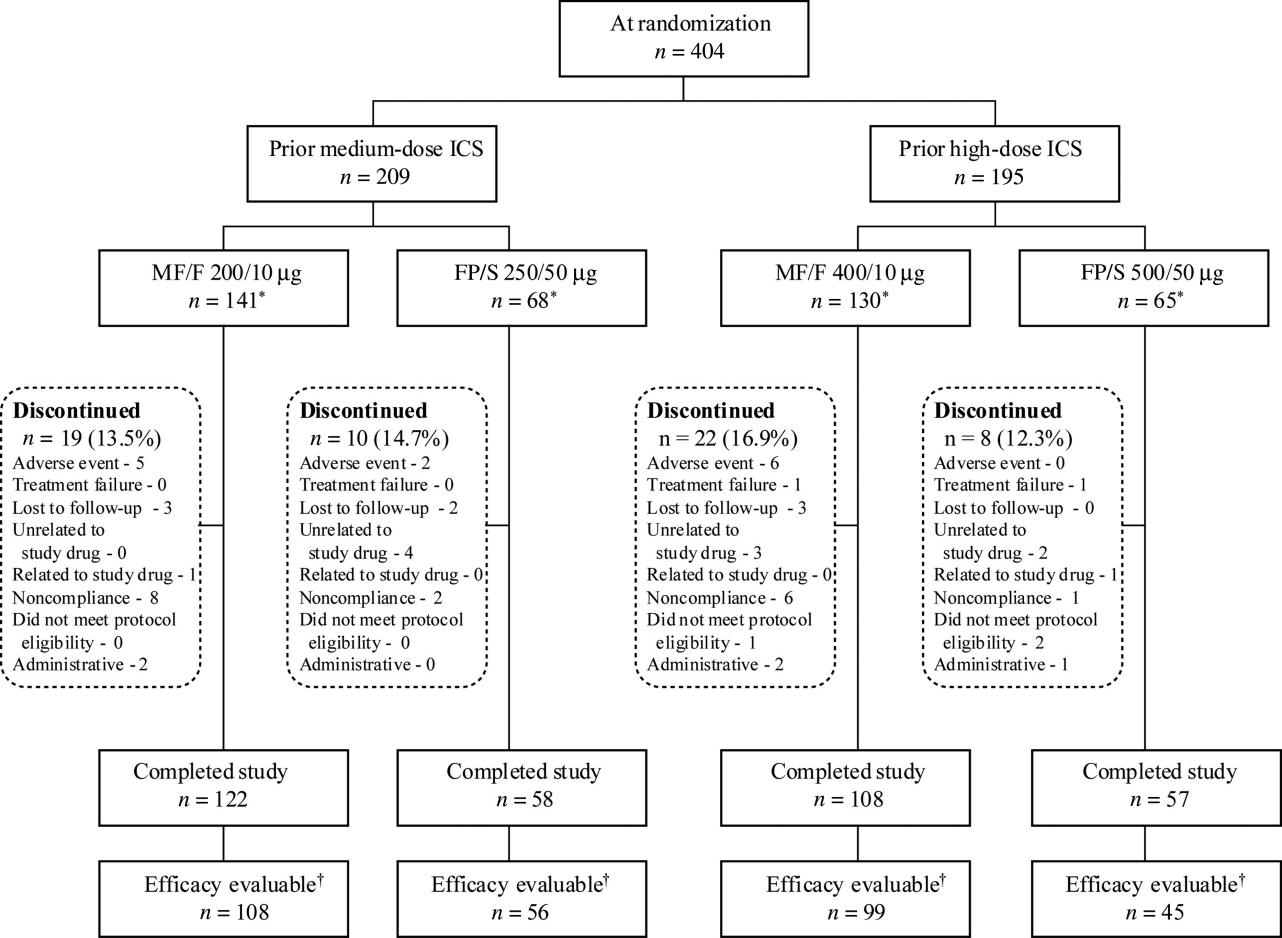
Patient disposition. ICS, inhaled corticosteroid; FP/S, fluticasone propionate/salmeterol combination; MF/F, mometasone furoate/formoterol. ‘Patient population used for safety analyses. tPatient population used for efficacy analyses, identified before database lock.

At screening, eligible patients were instructed to remain on their medication(s) until 24 hours before baseline visit and given salbutamol, a short-acting β_2_-agonist (SABA) as rescue medication. At baseline, patients were stratified (Figure 1) according to their previous ICS dose (medium or high) and randomized 2:1 to receive twice-daily (BID) treatment of two inhalations per dose of either MF/F (100/5 or 200/5 μg per inhalation) or FP/S (125/25 or 250/25 μg per inhalation). All study treatments were administered through MDIs. At the baseline visit, patients received instructions and training on the proper use of an MDI using a placebo training inhaler. Patients were not permitted to use spacer or holding chamber devices when administering study medication. Study drug compliance was assessed by monitoring pulmonary function, symptom score, SABA use, nocturnal awakenings, and clinical deterioration of asthma; patients considered nonadherent received instruction on necessary corrective measures.

### Study Population

Patients included in the study were 12 years or older, diagnosed with persistent asthma of >12 months, had a forced expiratory volume in 1 second (FEV_1_) >50% predicted values, received medium- or high-dose ICS with or without LAB A for >12 weeks before screening, and were on a stable regimen for >= 2 weeks before screening. Additional inclusion criteria were evidence of 2-reversibility (increase in FEV_1_ of >12% and >200 mL within 10–15 minutes of SABA use); normal electrocardiogram (ECG), clinical laboratory tests, and chest radiograph; and adequate contraceptive precautions for women of childbearing age.

Patients were excluded if they demonstrated a change >20% in FEV_1_; required use of >12 inhalations of SABA or two nebulized treatments with 2.5 mg salbutamol on 2 consecutive days at any time between the screening and baseline visits; experienced a clinically judged deterioration (deterioration resulting in emergency treatment, hospitalization, or treatment with additional asthma medication other than SABA); had intraocular pressure >22 mmHg in either eye, glaucoma, or evidence of cataract(s) at screening; was a current smoker (had smoked within the previous year) or ex-smoker (>10 pack-years); received emergency treatment for airway obstruction in the past 3 months; or suffered a respiratory infection within 2 weeks before screening.

A full medical history, including asthma, seasonal allergic rhinitis, and perennial allergic rhinitis, was obtained at baseline. Patients with a history of clinically significant medical illness or a disorder, that, in the judgment of the investigator, could interfere with the study or require treatment that might interfere with the study were not enrolled. Patients with conditions that were well controlled and stable (e.g., hypertension not requiring _β_-blockers) were allowed to participate if deemed appropriate per the investigator's judgment.

### Assessments of Safety and Efficacy

The primary objectives for the study were the number and percentage of all randomized patients who reported adverse events (AEs). A severe AE was any AE that caused inability to perform usual activities or significantly affected clinical status and warranted intervention. A serious AE (SAE) was any AE that was life-threatening or resulted in death, persistent or significant disability/incapacity, or required hospitalization. The secondary objective was an assessment of impact on hypothalamic-pituitary-adrenal (HPA) axis function as assayed by plasma cortisol 24-hour area under the curve (AUC_0–24 h_) measured at baseline, week 26, and week 52 at selected centers.

Additional safety evaluations were ophthalmologic tests (applanation tonometry and slit lamp examination with full dilatation) conducted at screening, week 26, and week 52 to assess the number and percentage of patients who had a change of at least 1 unit in lens opacities classification system (LOCS III) grade (22) or intraocular pressure >22 mmHg; physical examinations; clinical laboratory tests; and measurements of vital signs and ECG. Per protocol, a change of > 1 unit in LOCS III grade or intraocular pressure >22 mmHg was considered an SAE, and patients were to be discontinued from the study.

Clinical evaluations included pulmonary function tests by spirometry (FEV_1_, forced expiratory flow [FEF], forced vital capacity [FVC], percentage predicted FEV_1_) and peak flow meter measurements (morning and evening peak expiratory flow [PEF]); asthma symptom score, based on daily patient assessment of three asthma symptoms (wheezing, difficulty breathing, and cough) each scored on a scale of 0 (none) to 3 (very uncomfortable and interfered with most or all of normal daily activities/sleep); proportion of asthma-free days and nights (i.e., no SABA use, no asthma symptoms, 24-hour PEF variability less than 20% of baseline, no unscheduled visits to a medical facility, and no nocturnal awakening); proportion of days and nights without any SABA use; and clinically judged deterioration (asthma deterioration resulting in emergency treatment, hospitalization, or treatment with additional asthma medication other than SABA). FEV_1_, FVC, and FEF from 25% to 75% (FEF25_75%) were evaluated at baseline, each study visit, and endpoint; morning and evening PEF and asthma symptom scores were evaluated at baseline, each study month, and endpoint; proportions of days and nights that were asthma-free or SABA-free were evaluated at baseline and across the entire treatment period; incidences of asthma deterioration were recorded across the entire treatment period.

### Statistical Analyses

The sample size necessary to detect an event with a 2% incidence rate in at least 1 out of 100 patients with a 20% dropout rate was calculated to be 125. Results of AEs are tabulated by system organ class as defined by the Medical Dictionary for Regulatory Activities. Results of plasma cortisol AUC_0–24 h_ and ophthalmologic examinations are presented using descriptive statistics for each treatment group.

Analyses of lung function tests, asthma symptom scores, proportions of asthma-free days and nights, and days and nights without SABA use were based on randomized patients who met eligibility criteria (efficacy evaluable patients). Reasons for excluding patients from efficacy analyses were (1) failure to meet entrance criteria, (2) unacceptable baseline FEV_1_, (3) unacceptable concomitant medications, (4) insufficient medication washout, and (5) noncompliance with study treatment. These patients were identified after the completion of the study and before database lock. Because this study was not powered to determine differences in the outcome of these parameters between treatment groups, descriptive statistics are presented.

## Results

### Study Population

A total of 404 patients with persistent asthma were enrolled and randomized to receive MF/F 200/10 μg (*n* = 141) or FP/S 250/50 μg (*n* = 68) in the medium-dose stratum and MF/F 400/10 μg (*n* = 130) or FP/S 500/50 μg (*n* = 65) in the high-dose stratum, all administered BID through MDI. Overall, 345 patients (85%) completed the study and 59 patients (15%) discontinued treatment (Figure 1). Two common reasons for discontinuation were study noncompliance (*n* = 17, 4.2%) and AEs (*n* = 13, 3.2%), three of which were treatment-related. All randomized patients (*n* = 404) were included in the safety analyses, and all efficacy evaluable patients (*n* = 308) were included in the efficacy analyses.

Baseline demographics and disease characteristics were well matched between treatment groups within each stratum (Table 1). The majority of patients were between 18 and 65 years of age (82%), female (63%), and multiracial nonwhite (53%) with very few blacks or Asians. At entry, more patients had a body mass index of >25 kg/m^2^ in the high-dose stratum than the medium-dose stratum (63% vs. 44%, respectively). Mean ICS dose used before randomization was similar between MF/F and FP/S treatments within each stratum (861 vs. 905 μg/d in the medium-dose stratum, and 1431 vs. 1551 μg/d in the high-dose stratum; all beclomethasone equivalents); overall, 79% of patients did not use a concomitant LABA.

**Table 1:** Patient demographics and baseline characteristics

	Treatment group (MDI-administered; BID)				
	MF/F	FP/S	MF/F	FP/S	
	200/10 μg	250/50 μg	400/10 μg	500/50 μg	Total
Characteristics	(*n* = 141)	(*n* = 68)	(*n* = 130)	(*n* = 65)	(*n* = 404)
Age, mean (SD), years	32.7(15.2)	32.4(14.9)	39.3(14.5)	37.1(15.0)	35.5(15.2)
Sex, *n* (%)					
Women	92(65)	38(56)	86(66)	40(62)	256 (63)
Men	49 (35)	30 (44)	44 (34)	25(38)	148 (37)
Race, *n* (%)					
White	68 (48)	32 (49)	60(46)	32(49)	190(47)
Nonwhite (multiracial)	73(52)	38 (56)	70 (54)	33 (51)	214 (53)
Body mass index, mean (SD) (kg/m^2^)	25.78 (4.96)	25.56(5.51)	26.47 (4.27)	26.59 (5.54)	25.78 (4.96)
Asthma duration, mean (SD) (years)	15.32(11.92)	17.28 (12.5)	19.38(13.17)	18.10(12.30)	17.28(12.5)
Baseline FEY_1_ mean (SD) (L)	2.56(0.76)	2.53 (0.8)	2.31(0.72)	2.26(0.71)	2.42(0.76)
Baseline FEY_1_ mean (SD) (% predicted)	80.45(15.94)	78.14(16.26)	74.47 (15.80)	72.15(17.66)	76.78(16.49)
FEV_1_, mean (SD) (% reversibility)	21.28(9.54)	23.70(11.74)	24.05(11.68)	24.77 (13.86)	23.06(11.27)
Prior ICS use, *n* (%)*					
Beclomethasone	40(28)	15 (23)	28(22)	15(23)	97(24)
Budesonide	44(31)	24(35)	25(19)	13 (20)	106(26)
Ciclesonide	1(1)	1(1)	3(2)	0	5(1)
Fluticasone	38(27)	29 (32)	46(35)	24 (37)	129 (32)
Mometasone	1(1)	0	5(4)	1(2)	7(2)
ICS + LABA, *n* (%)*					
Budesonide/formoterol	3(2)	1(1)	1(1)	0	5(1)
Fluticasone/salmeterol	21 (15)	11 (16)	31 (24)	16 (25)	79 (20)
Prior tobacco use, *n* (%)	17(12)	4(6)	15(12)	9(14)	45(11)

* Patients could have used >1 ICS and/or ICS + LABA during the 3 months before randomization. BID, twice daily; FEV_1_, forced expiratory volume in 1 second; FP/S, fluticasone propionate/salmeterol; ICS, inhaled corticosteroid; LABA, long-acting β2-agonist; MDI, metered-dose inhaler; MF/F, mometasone furoate/formoterol.

### Safety

The majority of patients (83–88%) in each group completed the 52-week treatment period, thereby having sufficient exposure to the MF/F or FP/S dose to characterize their safety profiles. The number and percentage of patients reporting any AE in each group were as follows: MF/F 200/10 μg, *n* = 109 (77.3%); FP/S 250/50 μg, *n* = 56 (82.4%); MF/F 400/10 μg, *n* = 103 (79.2%); and FP/S 500/50 μg, *n* = 50 (76.9%). No noticeable differences in the nature or frequency of AEs were observed between groups (Table 2). The most common AE categories were infections and infestations; nervous system disorders; gastrointestinal disorders; and respiratory, thoracic, and mediastinal disorders. The majority of AEs were of mild to moderate severity. About one-third of AEs in each group were judged by investigators as likely related to treatment (MF/F 200/10 μg, 36.7%; FP/S 250/50 μg, 28.6%; MF/F 400/10 μg, 29.1%; and FP/S 500/50 μg, 26.0%). The percentage of patients reporting any treatment-related AEs was similar regardless of dose or study treatment: MF/F 200/10 μg, 28.4%; FP/S 250/50 μg, 23.5%; MF/F 400/10 μg, 23.1%; and FP/S 500/50 μg, 20.0%. Frequently reported treatment-related AEs (Table 3) in the two MF/F treatment groups were dysphonia (4.1%), headache (3.7%), tremor (2.2%), and aphthous stomatitis (1.5%). Oral candidiasis (1.1%) and pharyngitis (0.74%) were rare among all patients receiving MF/F. These results were similar to those observed in the two FP/S treatment groups; however, zero incidences of dysphonia or aphthous stomatitis were reported among patients receiving FP/S.

**Table 2:** Most common treatment-emergent adverse events reported by >5.0% of patients in any treatment group.

	Treatment group (MDI-administered; BID)				
	MF/F	FP/S	MF/F	FP/S	
	200/10 μg	250/50 μg	400/10 μg	500/50 μg	Total
MedDRA Classification, *n* (%)	*n* = 141)	*n* = 68)	*n* = 130)	*n* = 65)	n= 404)
Infections/ infestations	86(61.0)	46 (67.6)	76(58.5)	34 (52.3)	242 (59.9)
Nasopharyngitis	29 (20.6)	13(19.1)	21 (16.2)	8(12.3)	71(17.6)
Bronchitis	17(12.1)	14(20.6)	20(15.4)	7(10.8)	58(14.4)
Influenza	14 (9.9)	9(13.2)	13(10.0)	11(16.9)	47(11.6)
Pharyngitis	15(10.6)	9(13.2)	11(8.5)	9(13.2)11(8.5)	44(10.9)
Rhinitis	7 (5.0)	8(11.8)	7(5.4)	1(1.5)	23 (5.7)
URTI	5(1.5)	5(7.4)	5(3.8)	1(1.5)	16(4.0)
Sinusitis	7(5.0)	2 (2.9)	4(3.1)	1(1.5)	14(3.5)
Nervous system disorders	47(33.3)	20 (29.4)	36 (27.7)	13 (20.0)	116(28.7)
Headache	33 (23.3)	17(25.0)	31(23.8)	13 (20.0)	94(23.3)
Gastrointestinal disorders	35(24.8)	22(32.4)	30(23.1)	18(27.7)	105(26.0)
Abdominal pain	4(2.8)	3 (4.5)	6 (4.6)	5(7.7)	18(4.5)
Abdominal pain upper	3(2.1)	3 (4.4)	7(5.4)	3 (4.6)	16(4.0)
Aphthous stomatitis	3(2.1)	4 (5.9)	3 (2.3)	0	10(2.5)
Respiratory, thoracic, mediastinal disorders	42(29.8)	19(27.9)	28(21.5)	11(16.9)	100(24.8)
Rhinitis allergic	11(7.8)	6(8.8)	9 (6.9)	5(7.7)	31(7.7)
Pharyngolaryngeal pain	11(7.8)	2(2.9)	7(5.4)	1(1.5)	21 (5.2)
Dysphonia	7 (5.0)	5(7.4)	5(3.8)	2(3.1)	19(4.7)
Cough	8(5.7)	4(5.9)	2(1.5)	2(3.1)	16(4.0)
Musculoskeletal and connective tissue disorders	21(14.9)	13(19.1)	20(15.4)	11(16.9)	65(16.1)
Back pain	10(7.1)	7(10.3)	6(4.6)	3 (4.6)	26 (6.4)
Arthralgia	7(5.0)	3 (4.2)	4(3.1)	3 (4.6)	17 (4.2)
Muscle spasms	2(1.4)	2(2.9)	3 (2.3)	4 (6.2)	11(2.7)
General disorders and administrative site conditions	19(13.5)	9(13.2)	13(10.0)	5(7.7)	46(11.4)
Pyrexia	8(4.7)	4(5.9)	6(4.6)	1(1.5)	19(4.7)

BID, twice daily; FP/S, fluticasone propionate/salmeterol; MDI, metered-dose inhaler; MedDRA, Medical Dictionary for Regulatory Activities; MF/F, mometasone furoate/formoterol; URTI, upper respiratory tract infection.

**Table 3:** Most common treatment-related adverse events reported by >2.0% of patients in any treatment group.

	Treatment group (MDI-administered; BID)				
	MF/F	FP/S	MF/F	FP/S	
	200/10 μg	250/50 μg	400/10 μg	500/50 μg	Total
MedDRA Classification, *n* (%)	(*n* = 141)	(*n* = 68)	(*n* = 130)	(*n* = 65)	(*n* = 404)
Nervous system disorders	16(11.3)	4 (5.9)	6 (4.6)	2(3.1)	28 (6.9)
Headache	6 (4.3)	4 (5.9)	4(3.1)	1 (1.5)	15 (3.7)
Tremor	4 (2.0)	0	2(1.5)	2(3.1)	8 (2.0)
Infections/Infestations	7 (5.0)	5 (7.4)	8 (6.2)	5 (7.7)	25 (6.2)
Bronchitis	2(1.4)	2 (2.9)	3 (2.3)	1 (1.5)	8 (2.0)
Oral candidiasis	2(1.4)	1 (1.5)	1 (0.8)	2(3.1)	6(1.5)
Pharyngitis	2(1.4)	2 (2.9)	0	1 (1.5)	5 (1.2)
Respiratory, thoracic, mediastinal disorders	11 (7.8)	3 (4.4)	6 (4.6)	0	20 (5.0)
Dysphonia	7 (5.0)	0	4(3.1)	0	11 (2.7)
Gastrointestinal disorders	7 (5.0)	5 (7.4)	4(3.1)	2(3.1)	18 (4.5)
Aphthous stomatitis	3 (2.1)	0	1 (0.8)	0	4 (1.0)
Dysphagia	0	2 (2.9)	0	0	2 (0.5)
Musculoskeletal and connective tissue disorders	4 (2.8)	3 (4.4)	4(3.1)	3 (4.6)	14 (3.5)
Arthralgia	2(1.4)	3 (4.4)	1 (0.8)	1 (1.5)	7 (1.7)
Muscle spasms	1 (0.7)	1 (1.5)	2(1.5)	2(3.1)	6(1.5)
Eye disorders	3 (2.1)	1 (1.5)	5 (3.8)	0	9 (2.2)
Lens disorders	0	1 (1.5)	3 (2.3)	0	4(1.0)

BID, twice daily; FP/ /salmeterol; MDI, metered-dose inhaler; MedDRA, Medical Dictionary for Regulatory Activities; MF/F, mometasone furoate/formoterol.

A total of 21 patients (5.2%) reported severe or life-threatening AEs: MF/F 200/10 μg, *n* = 8 (5.7%); FP/S 250/50 μg, *n* = 4 (5.9%); MF/F 400/10 μg, *n* = 5 (3.8%); and FP/S 500/50 μg, *n* = 4 (6.2%). Only 2 of these 21 patients had severe or life-threatening AEs that were judged to be treatment related (1 patient with severe pneumonia and depressed level of consciousness in the MF/F 200/10 μg group and 1 patient with severe anxiety in the FP/S 500/10 μg group).

There were 21 patients who reported SAEs: MF/F 200/10 μg, *n* = 7 (4.9%); FP/S 250/50 μg, *n* = 4 (5.8%); MF/F 400/10 μg, *n* = 8 (6.1%); and FP/S 500/50 μg, *n* = 2 (3.1%). Most SAEs were unrelated to treatment, including two deaths (electrocution and gastric cancer). No asthma-related deaths or intubations occurred. Only 6 of these 21 patients had SAEs that were judged to be treatment related, including the patient with pneumonia and depressed level of consciousness mentioned previously.

The other five patients reported SAEs of mild to moderate eye disorders: 4 (3.1%) in the MF/F 400/10 μg group and 1 (1.5%) in the FP/S 250/50 μg group.

### Plasma Cortisol AUC_0–24 h_

**Figure 2:**
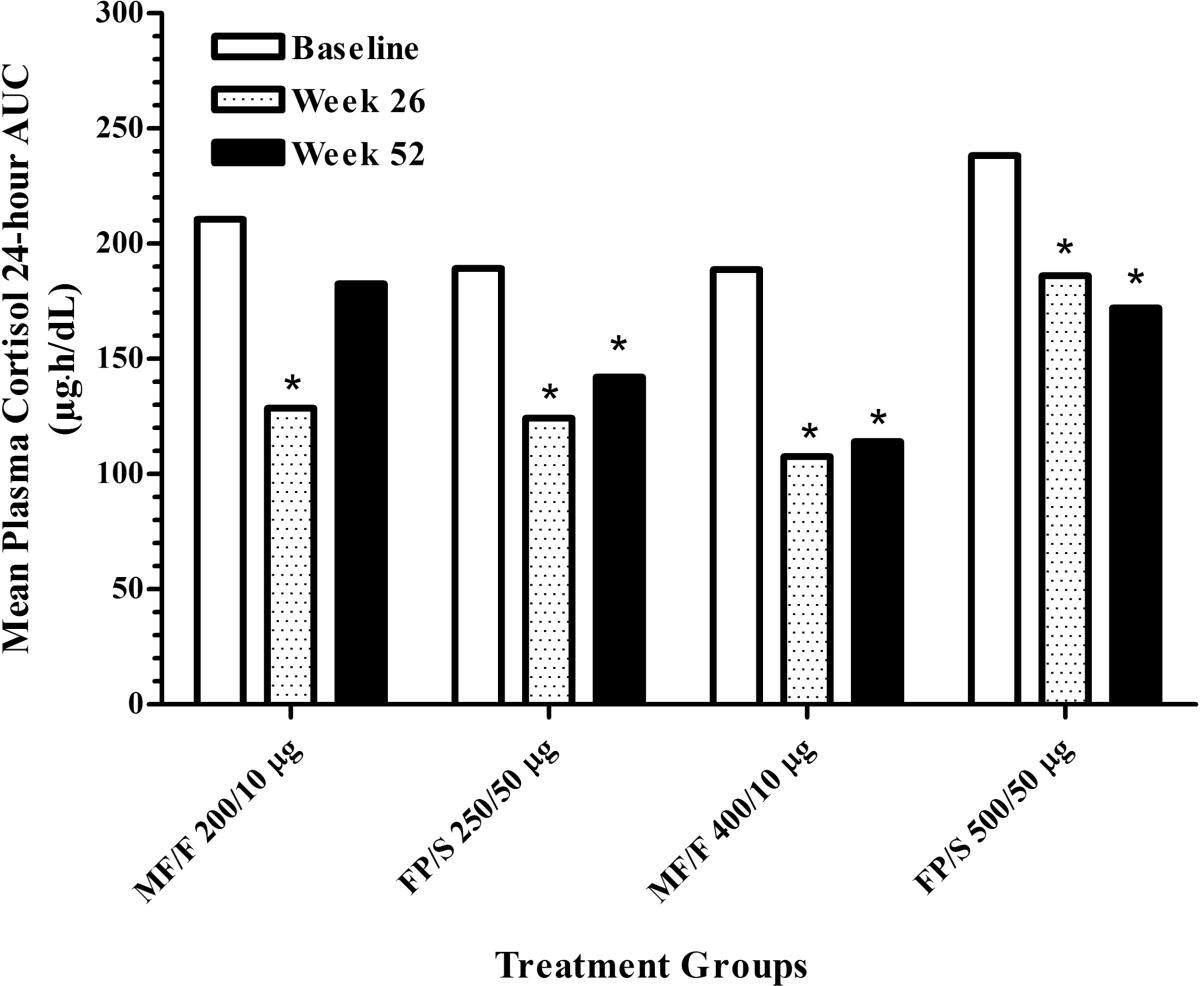
Mean plasma cortisol 24-hour AUC at baseline, week 26, and week 52. Patients who had both 0- and 24-hour measurements were included in the analysis. AUC, area under the curve; FP/S, fluticasone propionate/salmeterol; MF/F, mometasone furoate/formoterol. *p < .043 versus baseline within treatment groups.

The safety of long-term use of MF/F was further evaluated by measuring changes in plasma cortisol AUC_0–24 h_ in 57 patients (Figure 2). Mean baseline plasma cortisol AUC_0–24h_ levels were higher in the MF/F 200/10 μg and FP/S 500/50 μg groups (210.5 and 238.3 μg • h/dL, respectively) than in the MF/F 400/10 μg and FP/S 250/50 μg groups (188.8 and 189.2 μg • h/dL), which was likely because of high values found in three individuals (two in the MF/F and one in the FP/S groups). Compared with the baseline values, there were sustained statistically significant reductions in plasma cortisol AUC_0–24h_ in all treatment groups (*p* < .043) at weeks 26 and 52, with the exception of a nonsignificant reduction for FP/S 250/50 μg at week 52 (*p* = .076). At week 26, the extents of decreases were 37.5% for MF/F 200/10 μg, 28.8% for FP/S 250/50 μg, 33.3% for MF/F 400/10 μg, and 22.3% for FP/S 500/50 μg. At week 52, the corresponding decreases were 2.2%, 16.7%, 29.6%, and 32.2%.

### Ocular Changes

For all treatments, ophthalmologic examinations revealed a low incidence of ocular events as defined by the percentage of patients with a LOCS III grade change of at least 1 unit during the study: MF/F 200/10 μg, 3.5% (*n* = 5); FP/S 250/50 μg, 5.9% (*n* = 4); MF/F 400/10 μg, 3.8% (*n* = 5); and FP/S 500/50 μg, 1.5% (*n* = 1). Furthermore, there was no report of posterior subcapsu-lar cataracts in any treatment group. Of the 15 patients reporting ocular changes, 5 experienced changes that were considered by the investigator as being possibly related to study treatment (all considered SAEs): 3 with a lens disorder, 1 with ocular hypertension (all MF/F 400/10 μg), and 1 with reduced visual acuity (FP/S 250/50 μg). Treatment was discontinued for two of the patients with lens disorder and the patient with reduced visual acuity.

### Assessments of Lung Function, Asthma Symptoms, and Other Asthma-Related Parameters

At baseline, patients in the medium-dose stratum had higher FEV_1_ values (MF/F 200/10 μg, 2.65 L and FP/S 250/50 μg, 2.62 L) than those in the high-dose stratum (MF/F 400/10 μg, 2.31 L and FP/S 500/50 μg, 2.41 L), indicating less impairment in lung function in the medium-dose stratum. Analyses of pulmonary function tests revealed that patients showed improvements in lung function across all treatment groups as early as week 1. At week 52, FEV_1_ values increased on average by 10.7%, 15.0%, 11.7%, and 14.8% for MF/F 200/10 μg, FP/S 250/50 μg, MF/F 400/10 μg, and FP/S 500/50 μg, respectively. Similarly, other spirometry measures (percentage predicted FEV_1_, FVC, and FEF_25–75%_) demonstrated increases from baseline in all treatment groups (data not shown), providing further evidence for improved lung function. Measurements of PEF, which were taken twice daily by patients before study medications, showed mean percentage increases from baseline to month 12 in both morning PEF (MF/F 200/10 μg, 21.0%; FP/S 250/50 μg, 18.6%; MF/F 400/10 μg, 14.5%; FP/S 500/50 μg, 9.6%) and evening PEF (21.5%, 18.8%, 13.6%, 8.9%, respectively). Percentage increases from baseline were observed at every time point measured throughout the study, beginning with month 1.

**Figure 3:**
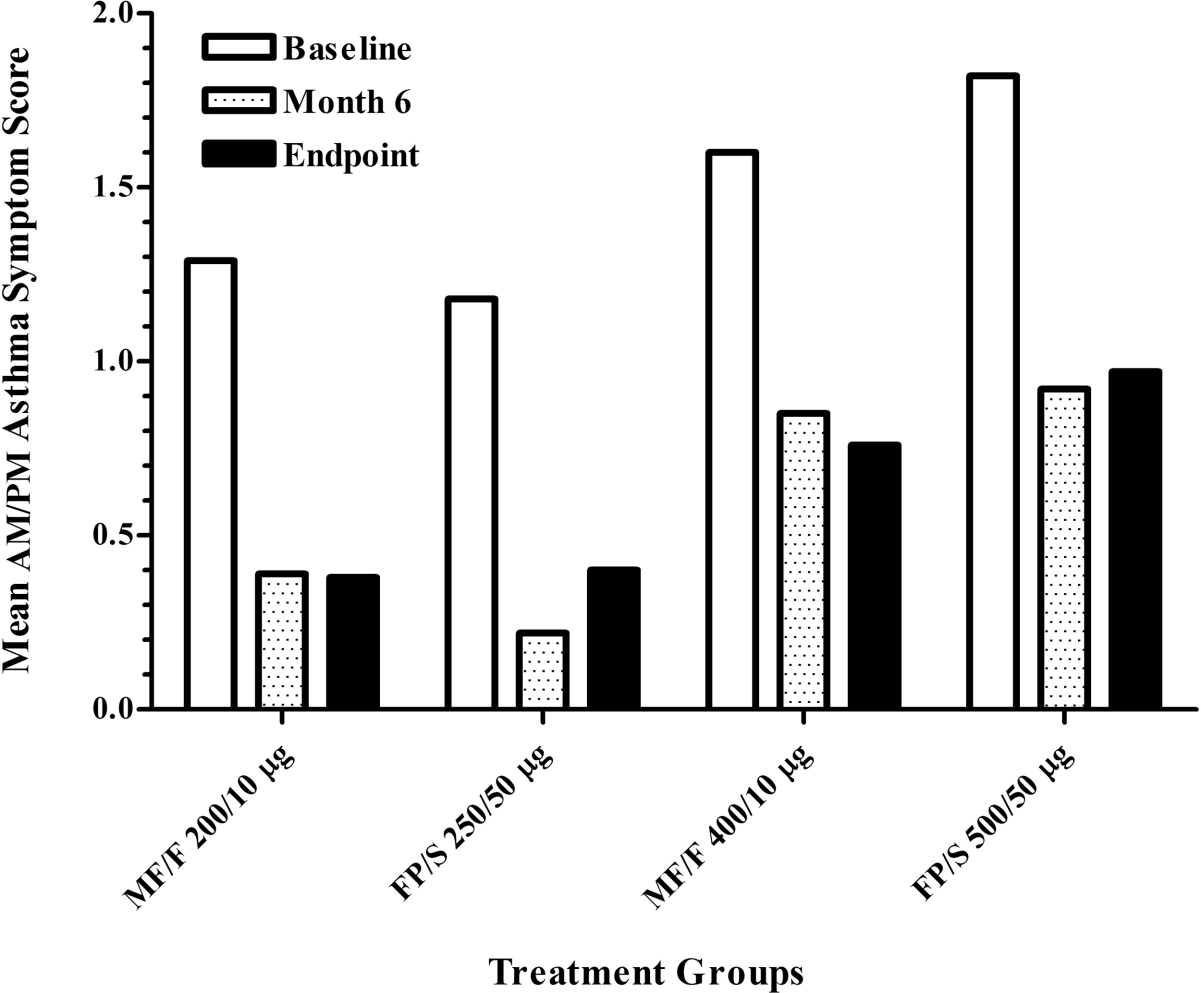
Improvement in mean 24-hour total asthma symptom scores. Total asthma symptom scores are the sum of wheezing, coughing, and difficulty breathing scores, based on a scale of 0 (none) to 3 (severe). FP/S, fluticasone propionate/salmeterol; MF/F, mometasone furoate/formoterol.

Patient-assessed mean 24-hour total asthma symptom scores were lower (i.e., improved) in the medium-dose stratum (MF/F 200/10 μg, 1.29; FP/S 250/50 μg, 1.18) than in the high-dose stratum (MF/F 400/10 μg, 1.60; FP/S 500/50 μg, 1.82) at baseline. All scores in all treatment groups were lower at each monthly visit during the study and at study endpoint compared with baseline (Figure 3). Absolute changes from baseline in mean 24-hour total asthma symptom scores at study end-point represented improvement of 63.9% with MF/F 200/10 μg, 45.0% with FP/S 250/50 μg, 34.5% with MF/F 400/10 μg, and 48.2% with FP/S 500/50 μg. Furthermore, proportion of asthma-free days and nights (i.e., no SABA use, no asthma symptoms, 24-hour PEF variability less than 20% of baseline, no unscheduled visits to a medical facility, and no nocturnal awakening) increased by 3.6-fold with MF/F 200/10 μg, 4.3-fold with FP/S 250/50 μg, 9.3-fold with MF/F 400/10 μg, and 3.4-fold with FP/S 500/50 μg. The proportions of days and nights free of SABA use increased by 47%, 65%, 78%, and 100% in the MF/F 200/10 μg, FP/S 250/50 μg, MF/F 400/10 μg, and FP/S 500/50 μg treatment groups, respectively.

During the study period, 56 randomized patients (13.9%) experienced a clinically judged deterioration resulting in emergency treatment, hospitalization, or treatment with additional asthma medications, but none of them were life-threatening. The percentage of patients with a clinically judged deterioration was similar between treatment groups within each stratum: 9.9% (MF/F 200/10 μg) versus 8.8% (FP/S 250/50 μg) in the medium-dose stratum and 17.7% (MF/F 400/10 μg) versus 20.0% (FP/S 500/50 μg) in the high-dose stratum.

## Discussion

This was the first study to assess the long-term safety of novel MF/F combination therapy in patients with persistent asthma. The study demonstrated that MDI-administered MF/F 200/10 and 400/10 μg BID were well tolerated and exhibited safety profiles similar to those of the individual components (12, 13, 18, 23) as well as of FP/S at equivalent doses (20, 21). There were no asthma-related deaths or intubations, and only two patients on MF/F discontinued the study owing to treatment-related AEs. The percentages of patients reporting at least 1 AE while receiving either MF/F treatment was comparable to those observed in two recent 52-week studies of budesonide/formoterol MDI 320/9 μg BID combination therapy in patients with asthma (24, 25). Because monocomponent treatment arms were not included in this study, direct comparisons of safety data between MF/F, MF, and F are not possible. However, data from two shorter studies, including a 26-week study (MF/F 200/10 μg, MF 200 μg, F 10 μg, and placebo treatment groups) (26) and a 12-week study (MF/F 200/10 μg, MF/F 400/10 μg, and MF 400 μg treatment groups) (27) indicated that no new signals were observed for MF/F compared with its monocomponents. Although dysphonia is a common AE associated with ICS monotherapy (28, 29), the incidence of dysphonia was low in this study. Similarly, the incidence of oral candidiasis was low. In addition, reports of tremor and muscle spasms, AEs commonly associated with LABA use (30,31), were very low among all patients treated with MF/F. No clinically abnormal changes in ECG measurements, vital signs, or plasma potassium levels were seen with MF/F treatment. Taken together, these data demonstrate that long-term use of MF/F at either dose raised no specific safety issues.

Because high doses of exogenous glucocorticoids can potentially suppress the production of endogenous corti-sol, HPA-axis suppression is often used to measure a systemic activity of corticosteroids (23, 28, 32, 33). Previous studies indicated minimal effects of MF on plasma and urinary cortisol levels at recommended doses; however, at higher doses a significant HPA-axis suppression was observed that was similar to the effects seen with high doses of FP (7, 29, 34, 35). In this study, there were statistically significant decreases from baseline in plasma cortisol AUC_0–24h_ for all treatment groups at week 26 and for three of the four treatment groups at week 52. There is no clear explanation for these decreases because the equivalent ICS doses of both study treatments were not higher than the patients’ previous ICS doses. One possible explanation for this is that patients’ compliance with medication improved once they were on study drug; however, data are not available to confirm this hypothesis. Although clinical relevance of these decreases could not be ascertained owing to lack of placebo group, the finding that the extent of decreases in plasma cortisol AUC_0–24 h_ in the MF/F treatment groups was similar to those seen in the FP/S groups suggests an acceptable safety profile of MF/F with regard to this systemic side effect at the doses tested.

This was the first study to measure effects of MF/F on potential ocular changes using LOCS III system. Overall, there were only five treatment-related ocular changes of mild to moderate intensity. More importantly, ocular changes associated with steroid treatment such as posterior subcapsular cataract and glaucoma were absent. This outcome is similar to the results reported in other 52-week studies in which only minor effects were seen in mean changes in LOCS III scores or intraocular pressure using other ICS treatment for asthma: ciclesonide and beclomethasone dipropionate in adult patients (36) and FP in pediatric patients (37).

Although this study was not designed to evaluate clinical benefits of MF/F, lung function tests and other asthma-related clinical outcomes were measured to ensure that MF/F treatment had no adverse impact on patients’ health. Study results showed that following the switch from prestudy treatment (medium- or high-dose ICS administered with or without LABA) to MF/F study treatment, patients experienced rapid improvements in respiratory function and asthma symptoms that were sustained throughout the 52-week MF/F treatment period. Furthermore, clinically judged deteriorations occurred infrequently in MF/F treatment groups and with similar frequency as in the comparative FP/S groups (i.e., within medium- and high-dose strata). Importantly, no case of clinically judged deterioration was considered life-threatening in this study. These clinical outcomes are consistent with the positive efficacy outcomes reported for long-term (52 weeks) FP/S and budesonide/formoterol studies of patients with persistent asthma (38, 39), although it is important to note that differences between these studies and our current study (e.g., dry powder inhalers vs. MDI devices, patient age range, dosing regimen) make it difficult to make direct comparisons.

Collectively, our findings confirm those of previous long-term ICS/LABA combination studies (38, 39) in which no new or unexpected safety concerns were observed compared with the collective safety histories of the individual monocomponents. However, further investigations are needed given the current concerns regarding the use of long-term LABA therapy (40).

The open-label design of this study is a potential limitation because it may bias the patient or observer. Furthermore, the clinical relevance of some evaluations in this study such as plasma cortisol and ocular change are difficult to ascertain because of the lack of a placebo control. Although a placebo comparator was not included in this study, use of the medium- and high-dose FP/S active-treatment arms did allow for direct comparison of MF/F and FP/S safety, tolerability, and efficacy outcomes based on dose stratum.

In conclusion, long-term (52 weeks) MF/F-MDI 200/10 and 400/10 μg BID treatments were well tolerated, with safety profiles similar to the known safety profiles of the individual MF and F components and to FP/S at equivalent doses. Furthermore, no new safety findings were detected during either MF/F treatment. Long-term MF/F treatments were also associated with rapid improvements in respiratory function and asthma symptoms, which were sustained over the 52-week treatment period. Taken together, these results suggest that MF/F 200/10 and 400/10 μg BID combination therapies delivered through an MDI are safe and well tolerated and present new and convenient options for the treatment of persistent asthma in adolescents and adults.

## APPENDIX 1

### Investigator List

Argentina: Enrique Casal, Buenos Aires; Hector Defranchi, Buenos Aires; Carlos G. Di Bartolo, Cuidad Autonoma de Buenos Aires; Alberto L. Dolmann, Florida; Jose E. Jares, Ramos Mejia; Jorge F. Maspero, Ciudad de Buenos Aires; Luisa B. Rey, Buenos Aires; Ahahi Yanez, Ciudad de Buenos Aires. Chile: Carlos Bisbal, Rancagua; Susana Munoz, Talcahuano; Tamara Soler, Santiago. Ecuador: Eduardo Castro, Quito; Ivan Cherrez, Guayaquil; Rene Cordova, Cuenca; Efrén Guerrero, Quito. Guatemala: Victor Chur, Gualtemala; Edgar Contreras, Guatemala; Jeremias Guerra, Guatemala; Gerardo Martinez, Guatemala. Mexico: Jesus J. Moran-Ochoa, Lomas de Sotelo, D.F.; Chavira M. Perez, Mexico D.F. Peru: Marco Camere, Lima; William Chavez, Lima; Octavio Cubas, Lima; Felix Efrain, Lima; Andres Pineiro, Lima; Danilo Salazar, Lima.
