# Corrigendum

**DOI:** 10.3109/02770903.2010.550726

**Published:** 2011-01-18

In the *Journal of Asthma* 2010; 47: 1106-1115 for the article “Long-term safety of mometasone furoate/formoterol combination for treatment of patients with persistent asthma,” the authors have noticed an error since publication. In the section *Plasma Cortisol AUC_0–24 h_*, the sentence that begins “Compared with the baseline values...” should indicate that the nonsignificant reduction refers to MF/F 200/10 rather than FP/S 250/50 in order to coincide with the data presented in the figure. The sentence should read as follows:

“Compared with the baseline values, there were sustained statistically significant reductions in plasma cortisol AUC_0–24 h_ in all treatment groups (*p* ≤ .043) at weeks 26 and 52, with the exception of a nonsignificant reduction for MF/F 200/10 µg at week 52 (*p* = .076).”

The authors apologize for this error.

